# Exploring consumers’ intention toward domestic energy-saving vehicles: Some insights from China

**DOI:** 10.3389/fpsyg.2022.927709

**Published:** 2022-09-06

**Authors:** Zi-Xu Wang, Amer Hamzah Bin Jantan, Ruo-Xi Wu, Yue Gong, Meng-Ru Cao, Philip Pong Weng Wong, Lei Wang

**Affiliations:** ^1^School of Continuing Education, Xuzhou University of Technology, Xuzhou, China; ^2^School of Management, City University Malaysia, Petaling Jaya, Malaysia; ^3^City Graduate School, City University Malaysia, Petaling Jaya, Malaysia; ^4^School of Physical Culture, Xuzhou University of Technology, Xuzhou, China; ^5^Department of Hospitality and Tourism, School of Management, Xuzhou University of Technology, Xuzhou, China; ^6^School of Hospitality, Sunway University, Bandar Sunway, Malaysia

**Keywords:** consumer ethnocentrism, perceived value, consumer knowledge, technology acceptance model, domestic energy-saving vehicles

## Abstract

Policies to promote the usage of energy-saving vehicles (EVs), such as electric vehicles and hybrids, were introduced and implemented in many countries due to increasing awareness of the potential benefits of such vehicles on environmental and energy conservation. However, despite consumers’ claims of their concerns and positive attitudes toward environmental issues, those claims have not been translated into energy-saving vehicles’ purchasing behavior. Prior studies neglected the interrelationship between consumer ethnocentrism (CE), perceived value (PV), and consumer knowledge (CK) in influencing consumer behavior, including pro-environmental behavior. This study examines the relationship between CE, PV, CK, perceived usefulness (PU), perceived ease of use (PEU), attitude and intention to purchase domestic energy-saving vehicles. A total of 396 completed questionnaires were collected through convenience sampling in Xuzhou, China. The survey data were subjected to descriptive analysis and analysis of variance using SPSS. In addition, confirmatory factor analysis and structural equation modeling (SEM) were utilized for the hypotheses testing. The results revealed that CE positively influenced PV and CK; PV and CK positively influenced PU and PEU. CK positively influenced PV, while PU and PEU positively influenced attitude and intention, and PEU was shown to influence PU. Furthermore, attitude was shown to significantly influence intention to purchase domestic energy-saving vehicles. Lastly, the theoretical and practical implications of the outcomes were discussed, including the limitations of the research.

## Introduction

As a result of globalization, trade wars between countries (e.g., United States against China, the United States against Russia) has intensified leading to increased animosity between nations and has evoked the revival of nationalism and patriotism among people of different nationalities to reject or avoid purchasing foreign-made products and services (Latif et al., 2019; Ortega-Egea and Garcia-de-Frutos, 2021). Specifically, the perceived political and economic threat of China is reflected in the call of western consumers to boycott Chinese-made products and vice versa (Ortega-Egea and Garcia-de-Frutos, 2021). In this context, when there are alternative domestic products or services relative to foreign ones, Chinese consumer ethnocentrism (CE) tendencies could be reinforced (Wang W. et al., 2018). According to Shimp and Sharma (1987), CE reflects social identity as it refers to a tendency to favor domestic products over a product of foreign origin. Individuals normally like to think positively about themselves and strive for a positive social identity since this may lead to higher self-esteem and psychological wellbeing (Glassner and Tajfel, 1985; Turner, 1999). In marketing literature, extensive cross-cultural studies have identified the role of CE on attitude and intention toward domestic products or services (Acikdilli et al., 2018; Yin et al., 2019; Danilwan et al., 2020; Raskovic et al., 2020).

Since 2000, the Chinese automotive market has grown at an average rate of more than 17.5% per year, which has been accompanied by serious environmental problems regarding energy security issues and ecological damage (Jiang et al., 2021). Thus, energy-saving vehicles (EVs) (e.g., electric vehicles and hybrid vehicles) are considered an effective alternative for sustainable urban transport as such vehicles can result in the reduction of negative impacts on the environment and the conservation of rare non-renewable energy sources (Liu et al., 2019). The lack of brand identity and core technology has prevented the domestic automotive industry from breaking the firm grip on the EVs industry by foreign firms. However, the technology gap between China and the foreign countries is not really that significant in the EVs industry (Jiang et al., 2021). In 2018, the sales of EVs in China reached 125.62 million, the majority of which were domestically manufactured EVs, making China the world’s largest EVs producing country by sales volume, with an increasing number of Chinese consumers converting to EVs in the future (Liu et al., 2021).

To promote EVs adoption, it is important to understand how consumers perceive EVs and what are the possible drivers and barriers to adoption (Singh et al., 2020). China as a developing country is still in its infancy when it comes to CE research (Makanyeza and du Toit, 2017), especially when it comes to research on CE about domestic EVs. Previous studies have examined the antecedents of the consumers’ intention to the adoption of EVs and usually regarded the properties of perceived value (PV) as major obstacles to the diffusion of EVs (Zhuge and Shao, 2019; Kumar and Alok, 2020; Akbarov, 2021). Other studies indicated that such consumption value-related barriers may not act directly on the purchase of EVs and such value-attitude-intention/behavior studies seem to be unable to explain the behavior comprehensively (Wang et al., 2022b). Specifically, the acceptance of EVs derives to a large extent, from the consumers’ psychological perception of EVs (Rezvani et al., 2015; Wang S. et al., 2018; Featherman et al., 2021). Consumers with more knowledge about EVs are more likely to consider purchasing and enhancing the PV of the adoption of EVs (Jaiswal et al., 2021a). However, past studies also demonstrated that there is a lack of general knowledge about EVs among consumers (Jaiswal et al., 2021a). For example, Krause et al. (2013) indicated in their study that two-thirds of their respondents had misunderstandings about the basic characteristics of EVs in the United States.

**FIGURE 1:**
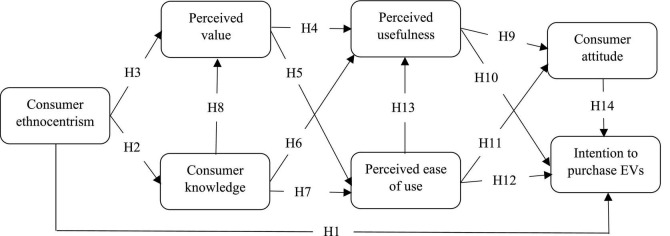
Conceptual research model.

Certain studies found that CE could significantly influence PV and consumer knowledge (CK) of domestic products and further influence the consumers’ desires to buy (Seitz and Roosen, 2015; Wang W. et al., 2018; Ortega-Egea and Garcia-de-Frutos, 2021). Nevertheless, previous studies have shown mixed and inconsistent results in these relationships. While some studies indicated that the purchase attitude toward domestic products could be influenced by CE and that consumers with high ethnocentric beliefs have moral difficulty in accepting foreign products (Qing et al., 2012; He and Wang, 2015), other studies showed that CE did not affect Chinese consumers’ attitude and intention toward domestic products (Tong and Li, 2013; Tsai et al., 2013). One reason for the contradictory findings could be due to the different roles of CE in different domestic product categories (Ding, 2017; Maksan et al., 2019), but more importantly, the impact of CE on the purchase intention of domestic products may vary depending on various aspects, such as the consumers’ psychological perceptions of the products (Akbarov, 2021). Moreover, the indirect role of CE on the purchase intention of domestic products and consumers’ psychological perceptions of domestic products are often ignored when CE is considered as an antecedent (Sharma et al., 1995; Shankarmahesh, 2006). In other words, the influence of CE on the product attributes may depend on other conditions, such as the effect of PV aspects (Wang W. et al., 2018). Considering that most previous studies in this domain focused on western consumers’ attitudes toward products from other western countries (Wang W. et al., 2018), this study seeks to extend the existing knowledge of the influence of CE on Chinese consumers’ attitudes and intentions toward purchasing domestic EVs with added variables of PV and CK based on the technology acceptance model (TAM) (Figure 1).

## Theoretical background and hypotheses development

### Technology acceptance model

TAM is often used to evaluate an individual’s attitude and intention toward purchasing and usage of new technology or innovative products (e.g., EVs) and services (Huang et al., 2021; Jaiswal et al., 2021b). TAM, which was developed and extended from various cognitive theories like the theory of reasoned action (Fishbein and Ajzen, 1977), theory of planned behavior (Ajzen, 1991), and rational choice theory (Goode, 1997; Venkatesh and Davis, 2000), has been employed by researchers to interpret user behavior toward the acceptance of new technologies (Davis, 1989; Jaiswal et al., 2021b). The classical TAM model measures an individual’s attitude and willingness to accept new technologies through two psychological factors: perceived usefulness (PU) and perceived ease of use (PEU) (Wang S. et al., 2018). However, researchers have argued that the classical TAM model only measures the advantageous belief-behavior relationships based on the technical attributes of the product (Wang and Hazen, 2016; Jaiswal et al., 2021b). Scholars have indicated that TAM could be more flexible to incorporate PV and CK to measure acceptance of technology (Yeo et al., 2017; Wang S. et al., 2018) in the context of EVs purchase to better understand the mechanisms that shape the progress of consumer acceptance of technology (Jaiswal et al., 2021a).

### Consumer ethnocentrism toward energy-saving vehicles

The behavior of individuals with cultural connotations on purchasing preferences based on the products’ country of origin can be considered a form of ethnocentrism (Stepchenkova et al., 2019). The human propensity to evaluate other cultures based on one’s in-group cultural norms and traditions over outgroups (Dursun et al., 2019), and the association of positive in-group attitudes and negative outgroup attitudes, is primarily generalized as outgroup negativity (Bizumic et al., 2009). CE is normally formed during early childhood socialization and is carried into adulthood with a few changes (Shimp and Sharma, 1987), and it leads individuals to unquestioningly embrace anyone or anything in their identifying group and to systematically reject all that falls outside of this group (Dursun et al., 2019).

In marketing, CE plays on the ideals held by purchasers on the appropriateness and morality of buying foreign-made products (Shimp and Sharma, 1987). Consumers with a high level of ethnocentrism resist purchasing imported products and consider foreign products as a threat which, from their point of view, destroys the domestic economy and causes unemployment (Tsai et al., 2013). Some researchers concluded that the consequences of CE stem from consumers’ overvaluation of domestic products and moral obligations (He and Wang, 2015). Thus, CE is an important determinant of purchase intention to adopt domestic products (Karoui and Khemakhem, 2019; Casado-Aranda et al., 2020), and a high level of CE can lead to a negative perception of foreign products and a perception that domestically produced products are of a higher quality (Xin and Seo, 2019). A study by Thomas et al. (2020) investigated the factors influencing consumer purchase behavior in India and their results showed a positive indirect effect of CE on consumers’ intention to purchase domestic cars and automobile through attitude. In another study, Dursun et al. (2019) explored how CE influence Russian consumers’ intention to buy Turkish products. Through their analysis of a convenience sampling of 241 respondents, the researchers indicated that CE has a strong impact on the intention to buy Turkish products. Thus, the following hypothesis was established:

H1: CE positively influences intention to purchase EVs.

Although a survey of the extensive literature revealed very little on the relationship between CE and CK constructs, a study by Seitz and Roosen (2015) on the impact of CE on Bavarian food knowledge showed that CE is negatively related to consumer products knowledge. It means that product knowledge of the consumers will decrease as their ethnocentrism increases. Thomas et al. (2020) demonstrated that more ethnocentric people are less open to foreign products and less willing to learn about foreign products. However, CK is strongly related to consumers’ experience, memories, familiarity and information search (Ricci et al., 2019). Following these findings, it is expected that in the knowledge structure of highly ethnocentric consumers, the knowledge share of domestic EVs will be higher than that of foreign EVs. Thus, the following hypothesis is proposed:

H2: CE positively influences CK to purchase EVs.

Ethnocentric consumers look with suspicion at non-domestic products that threaten their country’s economy, politics and even culture (Steenkamp et al., 2003). Ortega-Egea and Garcia-de-Frutos (2021) argued that CE can enhance psychological and performance risk perceptions and it is the strongest predictor of both types of perceived risks. Sometimes, ethnocentric consumers underrate foreign product properties which usually comprise quality, design, bundle, etc., while they overestimate the properties of native products or services (Wang W. et al., 2018). Such consumers generally are rewarding of residential items and judge them more favorably than foreign items, and hence, are less likely to purchase overseas products (Shimp and Sharma, 1987). It is expected that CE tendencies will trigger a high PV in choosing domestic EVs. In other words, when the level of CE is high, consumers are more willing to embrace domestic EVs even if they are not as good as foreign EVs in terms of quality and performance. Thus, the following hypothesis is proposed:

H3: CE positively influences PV to purchase EVs.

### Perceived value toward energy-saving vehicles

Consumer PV is considered a key component for clients to find the great deal while buying merchandise and services (Wang W. et al., 2018). As PV is inherent to the enjoyment of the use of a service or product, an individual’s PV cannot be decided objectively by the providers (Wang et al., 2022b). Previous studies indicated that PV is a multidimensional concept which includes social value, functional value, emotional value, epistemic value, and conditional value (Sheth et al., 1991). However, some studies confirmed that it is not always important to include all value sorts as most selection scenarios involve a smaller setting (Caber et al., 2020; Wang et al., 2022b). For conditional value, Caber et al. (2020) stated that it is not a value itself, but it displays the impact of a product’s application within the specific satiation and circumstance; meanwhile, for epistemic value, Rasoolimanesh et al. (2020) indicated that it could be integrated into emotional value as it is associated with hedonism, interest and cognition received from the goods or services. In addition, researchers have typically considered conditional value and epistemic value as being too transient (Rasoolimanesh et al., 2020). Thus, a universal and parsimonious assessment of a product or service has to be based on its functional terms of predicted performance, the satisfaction derived from emotional value and the social impacts of contact with different customers (Wang et al., 2022b).

Accordingly, social value, functional value, and emotional value are taken into consideration as multidimensional concepts which represent a patron’s PV in this study. The social value represents a person’s perceived benefit through the product’s affiliation with distinctive reference groups of clients (Sheth et al., 1991). It is derived from the ability of the products or services to enhance the consumers’ social self-concept (Rasoolimanesh et al., 2020) and connect with the customer’s self-image (Bautista et al., 2020) as interactions among clients of the said merchandise or service providers could have a profound impact on his/her buying experience (Wang et al., 2022b). As functional value is related to the realistic or technical blessings customers can obtain by the use of such merchandise and services (Hur et al., 2013), it is related to the perceived advantages of services or products’ utilitarian and physical performance (Carlson et al., 2019). Emotional value aims to satisfy the client’s mental desires for a service or product (Hur et al., 2013) because it displays the utility derived from affective emotions that a service or product generates (Rasoolimanesh et al., 2020). Emotional values have to be taken into consideration because the purchase of services or products can produce high-quality or terrible affective emotions (e.g., hedonic orientation and novelty) (Caber et al., 2020) although customers might not be searching for emotional blessings deliberately during the consumption process (Hur et al., 2013).

Certain research highlighted how PV affects consumers’ purchase intention amongst EVs buyers in China. For example, Zhuge et al. (2019) investigated the factors influencing the adoption of EVs in China and revealed that low vehicle price, vehicle usage, social influence and traffic restrictions facilitated the purchase of EVs. Berkeley et al. (2018) analyzed the barriers amongst drivers to adopt EVs in the United Kingdom and they concluded that a better understanding of cost saving, battery capacity, EVs technology, driving experience and vehicle design will stimulate consumers to buy EVs. Previous studies also indicated that some aspects of PV like good recharging time, the maximum speed of EVs, charging infrastructure availability and vehicle safety will encourage consumers to adopt EVs (Kumar and Alok, 2020; Asadi et al., 2021).

On the other hand, the importance of the role of the PV as a pro-environmental determinant variable of PU to adopt EVs has been well-documented in EVs literature. For instance, Wang et al. (2022b) explored the antecedent variables influencing consumers’ intention to purchase EVs in China. Results from that study showed that consumers believed that they could protect environmental resources by purchasing EVs due to their environmentally friendly nature and to impress others. A study by Smith et al. (2017) investigated the variables influencing EVs adoption in Australia, and concluded that consumer PV is a key predictor for buying EVs. Similarly, many other studies showed how individuals perceived environmental awareness value (e.g., global warming) of EVs can be used in their decision-making processes (Casals et al., 2016; Berkeley et al., 2018). Thus, the following hypotheses were proposed based on the above arguments:

H4: PV positively influences PU to purchase EVs.

H5: PV positively influences PEU to purchase EVs.

### Consumer knowledge toward energy-saving vehicles

CK is considered a major predictor that affects the consumers’ purchase decision process (Hadar et al., 2013). Consumers gather and form information in their memories as knowledge through various channels which can lead to preference for certain products (Oh and Abraham, 2016). Accordingly, CK is categorized into three components: subjective knowledge (i.e., patron notion of product and carrier knowledge), objective knowledge (i.e., what consumers truly know), and knowledge primarily based on experience (Wang Y. et al., 2020). This study concentrates on the analysis of the type of subjective knowledge which is more reliable for influencing actual behavior in the specific context of EVs (Huang et al., 2021). CK about domestic EVs is defined as consumers having a certain level of knowledge about the main features of domestic EVs and their product technological features, such as no carbon emissions, low noise, money saving, and fast charging (Tu and Yang, 2019). When purchasing domestic products, knowledgeable people often believe that buying locally made products may boost their economy and reduce unemployment (Han and Guo, 2018). Previous studies have shown that knowledge of environmentally friendly products leads people to show particular interest and awareness, indirectly or directly influencing individual pro-environmental behavior (Sheng et al., 2019; Jaiswal et al., 2020). Thus, consumers’ environmental knowledge about EVs is important for the promotion of domestic EVs (Degirmenci and Breitner, 2017). The performance of domestic EVs is evolving in all aspects, and therefore, consumers with a high level of technical product knowledge may allay fears and reduce potential risks, which may positively influence their behavioral willingness to adopt domestic EVs (Huang et al., 2021).

The significant positive relationship between CK and behavior has been confirmed by many previous studies in various settings (Jaiswal et al., 2020; Wang Y. et al., 2020). However, the indirect effect of CK on purchase behavior has rarely been investigated in the literature. Unfamiliarity with the knowledge of domestic EVs dominates consumer fears, which in turn, prevents consumers from perceiving the value of domestic EVs. When consumers will receive more knowledge about EVs, they will believe that EVs can be beneficial to their lives, environment or country and they will find it easier to use them (Wang and Hazen, 2016; Liu et al., 2018). In addition, many researchers have confirmed that CK of EVs has a widespread positive impact on consumer PV (Jaiswal et al., 2021a) as knowledgeable clients are more likely to value sustainability and are more ready to embrace green technology and pro-environmental products and services (Varah et al., 2021). Thus, the following hypotheses are proposed for testing:

H6: CK positively influences PU to purchase EVs.

H7: CK positively influences PEU to purchase EVs.

H8: CK positively influences PV to purchase EVs.

### Perceived usefulness to adopting energy-saving vehicles for protecting the environment

PU and PEU are crucial mental elements which have an impact on consumers’ recognition of progressive products (Wang S. et al., 2018; Cheng et al., 2019; Jaiswal et al., 2021b). They are important factors of the TAM which has been broadly used to recognize the consumer’s recognition of new technology or progressive products (Davis, 1989). Accordingly, PU is described as a person’s belief that utilizing a specific technology will enhance the productiveness in his/her life (Davis, 1989). People are more likely to develop positive attitudes and intentions to undertake a brand new technology if it has been verified to be a beneficial tool (Liu et al., 2018). Within the context of domestic EVs, sensible measurements of PU may be summarized as superior energy performance, environmentally friendly, longer driving distances and better quality of life (Wu et al., 2019).

Researchers believe that the promotion of EVs in marketing will produce potential benefits of transforming the existing transportation industry toward a cleaner and greener future (Lim et al., 2019). Certain studies on green marketing have shown how PU positively influences behaviors. For example, Higueras-Castillo et al. (2019) explored consumer attitude toward EVs in Spain. The results indicated a significant positive correlation between PU and attitude. In another study, Wang (2022) explored the variables influencing green hotel selection in China and showed that there is a significant causal path from PU to attitude, and subsequently, behavioral intention. In conclusion, individuals who believe they may be able to reduce environmental pollution problems through purchasing pro-environmental products or services (i.e., EVs) will be motivated to adopt favorable attitudes toward it. Thus, this study postulates the subsequent hypotheses:

H9: PU positively influences consumers’ attitude to purchase EVs.

H10: PU positively influences consumers’ intention to purchase EVs.

### Perceived ease of use toward energy-saving vehicles

PEU is defined as the degree to which an individual considers the effortlessness of using new technology or system (Davis, 1989). The fewer barriers customers encounter in the operation of new technologies, the more likely they are willing to accept them (Jaiswal et al., 2021b). In the context of technological innovation toward domestic EVs, PEU as a subjective judgment of EVs technology can enhance consumers’ psychological and physical comfort, and consequently, positively influences consumer attitudes toward the technology offering and their intention to adopt it (Davis, 1989; Wu et al., 2019; Akbarov, 2021). Certain scholars noted that PEU can influence consumer intentions to choose EVs technology directly, or indirectly through PU, as new technologies are only considered useful if they are easily accessible to people (Wang et al., 2017; Wang Y. et al., 2020). Jaiswal et al. (2021a) applied TAM to consumers’ intention toward EVs in India and revealed a positive direct effect of PEU on consumers’ intention, whereas PEU positively influences PU to adopt EVs. Therefore, based on the arguments above, the following hypotheses are proposed:

H11: PEU positively influences consumers’ attitude to purchase EVs.

H12: PEU positively influences consumers’ intention to purchase EVs.

H13: PEU positively influences PU to purchase EVs.

### Consumers’ attitude toward intention to purchase energy-saving vehicles

According to Davis (1989), the adoption of new technology is determined by behavioral intentions which are a combination of an individual’s attitude and PU. In various behavior-orientation theories (e.g., theory of reasoned action, theory of planned behavior, value-attitude-behavior model), behavioral intention is highly predictable by attitude (Wang L. et al., 2020; Wang et al., 2022a). As Ajzen (1989) has argued, a person’s attitude may influence the response to a stimulant, and he or she who holds a favorable attitude toward action will be more inclined to perform a particular behavior (Yeo et al., 2017). Certain studies showed how attitude influences consumer intention toward a given behavior; for example, Wang et al. (2022b) revealed that attitude had the strongest predictive capacity on intention toward purchasing EVs. Another study by Lim et al. (2019) found a positive relationship between attitude and intention to adopt EVs in Malaysia, while the positive evaluation of EVs determines intention to purchase EVs (Jaiswal et al., 2021b). Thus, the following hypothesis was developed for testing:

H14: Attitude positively influences intention to purchase EVs.

## Research methods

### Data collection

This study is a descriptive one using a cross-sectional approach with the population being respondents from Xuzhou, China, from 1 January to 28 February 2022. According to Ouyang et al. (2020), China’s first-tier cities such as Beijing, Shanghai, Guangzhou and Shenzhen are already saturated with cars. The sale of EVs has been slow to grow despite the government’s introduction of certain incentives to stimulate purchases of EVs. In contrast, there is still room for EV sales to grow in second-tier cities, such as Xuzhou. The mall intercept method was adopted for this study as it provides a high response rate (Maksan et al., 2019), and also because of the concentration of various brands of auto 4S shops or auto exhibitions that are located within shopping malls in China. The target respondents were potential customers who went into 4S car shops and exhibitions for acquiring information on EVs in shopping malls. The sample size was set at 500 and a gift was given for completing the questionnaire to improve the response rate. Overall, a total sample of 396 respondents participated in the survey resulting in an overall response rate of 79.2%.

### Measures

A self-administered and close-ended questionnaire with validated measurement scales was utilized in this study. Back translation is the most commonly utilized translation method applied in research (Malhotra and Birks, 2007), and to guarantee the translation quality of the questionnaire, there should be at least three bilingual experts involved in the back-to-back translation process (Wang et al., 2022b). Thus, the questionnaire was initially translated into Chinese by the first two translators, then back-translated to English by the second team of translators, after which, the researcher met with the translation team to discuss any differences found in the back translation. To validate the conceptual framework and avoid any potential confusion and inaccuracies, a pilot test was conducted with 30 respondents (Eldridge et al., 2016). The questionnaire was divided into three sections to gather empirical data. The first section focused on the added TAM variables: CE, CK and PV, where seven items related to CE were adapted from Akbarov (2021) and Lee H.-M. et al. (2021); three items related to CK were modified from Ullah et al. (2018) and six items belonging to PV were adapted from Wang et al. (2022b). The second section focused on the components of TAM: PU, PEU, attitude and intention. Three items belonging to PEU were adapted from Wu et al. (2019); five items belonging to PU were adapted from Globisch et al. (2018) and Huang et al. (2021); five items belonging to attitude were adapted from Asadi et al. (2021) and four items belonging to intention were adapted by Han et al. (2017) and He et al. (2018). The last section elicited relevant demographic characteristics.

### Common method bias

Common method bias (CMB) is a prevalent issue in survey studies (Hulland et al., 2018). To reduce CMB in this study, all questionnaire items have been checked by field experts to ensure respondents’ understanding, and the respondents were guaranteed privacy and confidentiality with their answers and personal information. The measures in the questionnaire used multiple scale types, including bipolar, differential, semantic and Likert scales. In addition, Podsakoff et al. (2003) indicated that a common latent factor variable can be used to examine CMB. A latent variable was included in the CFA model by connecting it to all variables, and the standardized regression evaluated the new model before comparing it with the original model which showed similar results after comparison. Lastly, Harman’s single factor test was used to test the impact of CMB. The results showed an exploratory factor analysis with a single factor accumulation of 46.045%, indicating CMB is not a significant issue for this study.

## Data analysis and results

The Statistic Package for Social Science (SPSS) version 19 was utilized for the descriptive statistics of the study. Confirmatory factor analysis (CFA) and structural equation modeling (SEM) tests were performed with AMOS. Covariance-based application (i.e., CB-SEM) is based on the common variance derived from the covariances between all constructs in the structural model, which determines how well the model can estimate the covariance matrix for the sample data with the ultimate goal of confirming the theory (Hair et al., 2014). In contrast, the main goal of variance-based application (e.g., PLS-SEM) is to minimize unexplained variance in the dependent variables, and it is well suited for analyzing predictive and complex models with a large number of variables and relationships (Hair et al., 2019). Thus, the CB-based CFA and SEM were utilized for this study to explore and describe the effect of various antecedent variables on consumers’ EVs purchase behavior based on the TAM model.

### Descriptive analysis

A total of 396 usable questionnaires were obtained for analysis. Table 1 displays detailed demographic information. The respondents comprised 74.5% male and 25.5% female; 61.6% of the total respondents were aged between 18 and 30, and most of the respondents (31.8%) reported monthly income from CNY3,001-4,500. There were about 51.8% of respondents who had completed a 4-year bachelor’s degree. The distributed data are normal when the skewness ranges from –2 to + 2, while the kurtosis ranges from –7 to + 7, exhibiting a stronger deviation from normality (Byrne, 2016). The results indicated that the skewness ranged from –1.961 to –0.377, whereas kurtosis ranged from –1.434 to 4.071, thus indicating normality. The Kaiser-Meyer-Olkin (KMO) and Bartlett’s test of sphericity showed that the KMO value was 0.948, *p* < 0.001, which reflects sampling adequacy.

**TABLE 1:** Sample characteristics (*N* = 396).

Items	Characteristic	Frequency	Percentage (%)
Gender	Male Female	295 101	74.5% 25.5%
Age (Year)	Below 18 18–30 31–45 46–60 Above 61	49 244 71 23 9	12.4% 61.6% 17.9% 5.8% 2.3%
Income	Below 1,700 yuan 1,701–3,000 yuan 3,001–4,500 yuan 4,501–6,000 yuan Above 6,001 yuan	39 89 126 76 66	9.8% 22.5% 31.8% 19.2% 16.7%
Educational level	Middle or high school Diploma (3-years) Bachelor (4-years) Master and above	51 98 205 45	12.1% 24.7% 51.8% 11.4%

### Measurement model test

Cronbach’s alpha value of equal to or over 0.7 is considered satisfactory for the testing of internal reliability (Hair et al., 2010). Factor loadings ought to surpass 0.5 and ideally be higher than 0.7 (Hair et al., 2010). High-factor loadings indicated that the factor extracted sufficient variance from that variable (Hulland et al., 2018), and the factor with low loadings was dropped (i.e., PV4, PV5, attittude3, CE4). For this study, all Cronbach’s alpha values ranged from 0.823 to 0.97. For testing the convergent validity of the measurement model, composite reliability (CR) of over 0.7 and the average variance extracted (AVE) value ought to be higher than 0.5 as recommended (Hair et al., 2010). Based on Table 2, convergent validity was established. In addition, the discriminate validity of the measurement model can be determined when both the average shared squared variance (ASV) and maximum shared squared variance (MSV) are less than AVE (Byrne, 2016), and the correlation coefficient between diverse constructs is lower than 0.9 (Meyers et al., 2016). As shown in Table 3, there were no correlation coefficients between variables higher than 0.9 and each construct’s AVE was higher than the MSV and ASV. Furthermore, the heterotrait-monotrait ratio of correlations (HTMT) was considered to assess discriminate validity. Table 4 shows that all constructs’ threshold values for HTMT were less than 0.9 (Henseler et al., 2015). Thus, both the Fornell-Larcker criterion and the HTMT test value indicate that discriminate validity was established.

**TABLE 2:** Convergent validity for measurement model.

Constructs (Cronbach’s alpha)	Items	Item loadings	CR	AVE
Perceived ease of use (α = 0.954)	PEU1. I think it would be easy to use domestic EVs PEU2. I think it would be easy for me to drive domestic EVs to anywhere I want PEU3. I think it would be easy for me to interact with domestic EVs	0.960 0.955 0.895	0.956	0.878
Perceived usefulness (α = 0.951)	PU1. Domestic EVs can improve my travel efficiency and improve my living quality PU2. Domestic EVs are useful to reduce my household expenditures on transportation PU3. Domestic EVs can relive my physical and mental fatigue and benefit my health PU4. Domestic EVs are useful to reduce carbon emission and alleviate the energy shortage problems PU5. Domestic EVs perform the task for which they were acquired	0.894 0.898 0.890 0.912 0.875	0.952	0.779
Intention (α = 0.943)	Inten1. I am willing to purchase a domestic EVs when purchasing a vehicle in the near future Inten2. I am willing to accept intelligent EVs when seeing more people using domestic EVs Inten3. I would like to recommend my friends to purchase domestic EVs Inten4. I will make an effort to purchase a EVs when purchasing a vehicle in the near future	0.907 0.904 0.882 0.899	0.943	0.806
Attitude (α = 0.910)	Purchasing domestic EVs for me: ATT1. Interesting ATT2. Pleasure ATT4. Positive ATT5. Funny	0.898 0.884 0.753 0.706	0.886	0.663
Consumer knowledge (α = 0.970)	CK1. I know that domestic EVs technology can assist with driving CK2. I know the technological performance (e.g., charging time, acceleration, driving comfort, driving range) of domestic EVs CK3. I know the technological advantages of domestic EVs over gasoline vehicles	0.950 0.957 0.962	0.970	0.915
Perceived value (α = 0.823)	PV1. EVs get good mileage and preserved some traditional facets (functional value) PV2. The EVs staffs were friendly, courteous and able to converse well (functional value) PV3. Drive EVs is exciting and make me enjoyable (emotional value) PV6. Purchasing EVs is a smart choice and I will feel proud of my EVs (social value)	0.714 0.710 0.760 0.696	0.812	0.519
Consumer ethnocentrism (α = 0.919)	CE1. Chinese products first, last and foremost CE2. Chinese should always buy Chinese products instead of imports CE3. It is always best to purchase Chinese products CE5. There should be very little trading or purchasing of products from other countries unless out of necessity CE6. We should purchase products manufactured in China instead of letting other countries get rich off us CE7. Chinese consumer who purchase products made in other countries are responsible for putting their fellow Chinese out of work	0.924 0.865 0.816 0.795 0.657 0.664	0.909	0.629

**TABLE 3:** Discriminate validity for measurement model (Fornell-Larcker criterion).

Item	AVE	MSV	ASV	1	2	3	4	5	6	7
1. Consumer knowledge	0.915	0.593	0.368	***0.956***						
2. Perceived usefulness	0.799	0.771	0.444	0.770	***0.894***					
3. Perceived ease of use	0.878	0.542	0.328	0.527	0.604	***0.937***				
4. Attitude	0.663	0.584	0.434	0.599	0.672	0.579	***0.814***			
5. Intention	0.806	0.771	0.480	0.765	0.878	0.736	0.704	***0.898***		
6. Perceived value	0.519	0.378	0.260	0.444	0.482	0.416	0.615	0.484	***0.720***	
7. Consumer ethnocentrism	0.629	0.584	0.316	0.441	0.499	0.522	0.764	0.505	0.585	***0.793***

Italic bold values denote the square root of AVE.

**TABLE 4:** Discriminate validity for measurement model (HTMT test).

Item	1	2	3	4	5	6	7
1. Perceived usefulness	1.000						
2. Perceived ease of use	0.610	1.000					
3. Attitude	0.684	0.606	1.000				
4. Intention	0.874	0.747	0.720	1.000			
5. Perceived value	0.490	0.414	0.628	0.489	1.000		
6. Consumer knowledge	0.768	0.538	0.603	0.762	0.448	1.000	
7. Consumer ethnocentrism	0.512	0.539	0.849	0.551	0.603	0.439	1.000

The next step ensued with the assessment of measurement model fit. According to Hu and Bentler (1999), the assessment of the overall goodness-o- fit and the estimation of parameters of the hypothesized models are used to determine if the models tested should be accepted or rejected and the measures are based on fitting the model to sample moments. In other words, the goodness-of-fit test is to compare the observed covariance matrix to the one estimated on the assumption that the model being tested is reliable. The results of the model fit indices showed that CMIN = 1293.411, DF = 351, *p* < 0.001, CMIN/DF = 3.685, SRMR = 0.059, GFI = 0.812, AGFI = 0.767, PGFI = 0.665, IFI = 0.923, TLI = 0.91, CFI = 0.923, PNFI = 0.776, PCFI = 0.798, RMSEA = 0.082 demonstrated adequate model fit.

### Structural equation modeling test

**FIGURE 2:**
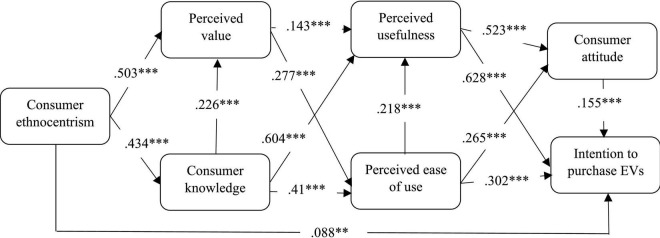
The structural model outcomes. ^**^p < 0.01, ^***^p < 0.001, and critical ratio > 1.96.

For the assessment of the structural model and testing of the proposed hypotheses, SEM was performed. The overall goodness-of-fit reported CMIN = 1516.968, DF = 358, *p* < 0.001, CMIN/DF = 4.237, AGFI = 0.758, PGFI = 0.659, IFI = 0.905, CFI = 0.905, PNFI = 0.775, PCFI = 0.798, RMSEA = 0.091. In other words, an adequate model fit was determined and the outcomes are tabulated in Figure 2 and Table 5, accordingly.

**TABLE 5:** Structural relationships and hypotheses testing.

Items	Parameter	Estimate	*P*-value	C.R.	S.E.	Decision
H1	Consumer ethnocentrism– > Intention	0.088	0.001	3.257	0.028	Supported
H2	Consumer ethnocentrism– > consumer knowledge	0.434	***	8.236	0.052	Supported
H3	Consumer ethnocentrism– > Perceived value	0.503	***	7.896	0.050	Supported
H4	Perceived value– > perceived usefulness	0.143	***	3.382	0.050	Supported
H5	Perceived value– > Perceived ease of use	0.277	***	4.951	0.095	Supported
H6	Consumer knowledge– > perceived usefulness	0.604	***	2.436	0.040	Supported
H7	Consumer knowledge– > Perceived ease of use	0.410	***	8.034	0.068	Supported
H8	Consumer knowledge– > Perceived value	0.226	***	13.945	0.040	Supported
H9	Perceived usefulness– > Attitude	0.523	***	9.598	0.058	Supported
H10	Perceived usefulness– > Intention	0.628	***	14.364	0.049	Supported
H11	Perceived ease of use– > Attitude	0.265	***	5.093	0.039	Supported
H12	Perceived ease of use– > Intention	0.302	***	8.828	0.027	Supported
H13	Perceived ease of use– > Perceived usefulness	0.218	***	5.317	0.029	Supported
H14	Attitude– > Intention	0.155	***	4.075	0.040	Supported

C.R., Critical ratio; S.E., Standard error. ***p < 0.001.

## Conclusion and discussion

Focusing on the factors that influence the purchasing of EVs in China, the extended TAM model that incorporated CE, CK and PV was empirically tested. According to Wang and Wong (2021), a path coefficient of below 0.1 means a small effect; a path coefficient of about 0.3 implies a moderate effect; and a path coefficient of 0.5 and above shows a large effect. The obtained results from this study indicated that CE positively influenced intention with a small effect (β = 0.088, *p* < 0.01); thus, H1 was supported. CE positively influenced CK with a moderate effect (β = 0.434, *p* < 0.001); thus, H2 was supported. CE and PV were positively corrected with a large effect (β = 0.503, *p* < 0.001); thus, H3 was supported. The results indicate that PV moderately positively influenced PU (β = 0.143, *p* < 0.001) and PEU (β = 0.277, *p* < 0.001); thus, H4 and H5 were supported. CK had a major influence on PU (β = 0.604, *p* < 0.001); thus, H6 was supported. CK also positively moderately influenced PEU (β = 0.41, *p* < 0.001) and PV (β = 0.226, *p* < 0.001); thus, H7 and H8 were supported. PU and attitude were positively correlated with large effects (β = 0.523, *p* < 0.001); thus, H8 was supported. PU and intention were positively correlated with large effects (β = 0.628, *p* < 0.001); thus, H10 was supported. The obtained results showed that PEU positively influenced attitude with moderate effects (β = 0.265, *p* < 0.001), intention with moderate effects (β = 0.302, *p* < 0.001), and PU with moderate effects (β = 0.218, *p* < 0.001), thus, H11, H12, and H13 were supported. The outcomes indicate that attitude and intention were positively correlated with moderate effects (β = 0.155, *p* < 0.001); thus, H14 was supported.

Thomas et al. (2020) mentioned that more ethnocentric people are less open to foreign products and less willing to learn about foreign products. Conversely, it is expected that high ethnocentric consumers are more likely to accept local products and more willing to learn about local products or services than about foreign products or services. The results showed a direct link between CE and CK about EVs. Wang W. et al. (2018) stated that ethnocentric consumers usually underestimate foreign product attributes (e.g., quality, design, package), but they have an overestimation of the value toward local products or services. Findings from this study indicated that CE positively and significantly influenced consumer PV about EVs. The above results demonstrate that consumers who perceived Chinese products and manufacturers favorably and are willing to purchase local products or services instead of import products or services, are more willing to acquire knowledge about local products (i.e., domestic EVs), as well as to recognize the unique attributes of such products or services.

PV has been proven to be a significant determinant that led to consumers’ decision-making processes in past studies (Caber et al., 2020; Rasoolimanesh et al., 2020; Wang et al., 2022b). The outcomes of this study indicated that PV had a positive influence on PU and PEU. This shows that consumers perceived that functional value aspects (e.g., EVs have good mileage and keep some traditional vehicle characteristics; EVs providers’ friendly interaction with customers); emotional value aspects (e.g., driving EVs are exciting and enjoyable) and social value aspects (e.g., purchasing EVs is a wise decision and they would be proud of purchasing them) significantly influenced their perceptions about purchasing and driving EVs as an enjoyable and beneficial activity for them.

The results of this study indicated that CK positively influences PU and PEU. As Jaiswal et al. (2021a) suggested, knowledge of EVs has a significant positive effect on consumer PV as more knowledgeable customers will more easily recognize the benefit of purchasing green technology and pro-environmental products and services (Varah et al., 2021). The results of this study show that there is a positive relationship between CK and PV. All of the above results mean that consumers who have more subjective knowledge about EVs (e.g., charging time, acceleration, oil consumption, comfort and other advantage attributes compared with traditional fuel vehicles) are more likely to perceive EVs as a useful, easy to drive, value-dominated and environmentally friendly mode of transport.

Many studies have proven that PU has a significant positive influence on attitude (Liu et al., 2018; Wu et al., 2019) and intention (Thøgersen, 2018; Wang S. et al., 2018).

This study showed that there is a positive relationship between PU and attitude toward purchasing EVs, indicating that consumers believe that EVs are reliable and environmentally friendly. EVs can provide savings in household expenditures, and more importantly, benefiting and protecting the environment. In other words, consumers believe EVs can improve their quality of life, reduce transportation problems, increase their level of health, and reduce energy consumption without sacrificing their living standards.

Previous studies showed that there is a positive significant relationship between PEU and attitude and intention (Wu et al., 2019; Jaiswal et al., 2021a). The results suggest that PEU positively influences attitude and intention. This means that consumers who think that they can easily buy and drive domestic EVs is a key determinant for their purchasing attitude and intention. Certain researchers argued that PEU plays a complicated role in TAM due to PEU also had a significant influence on PU in past studies (Wang et al., 2017; Wang Y. et al., 2020) which in contrast with studies dependent on the models originating with the theoretical framework of the TAM. The results showed that PEU positively influenced PU. It means that consumers who perceived EVs are easy to drive will result in higher perceptions of the usefulness of purchasing EVs. The results of this study stand in line with many studies which state that attitude emphatically impacts the intention to make a purchasing behavior (Lee C.-K. et al., 2021; Wang et al., 2021). Consumers who have evaluated the product features of EVs will perceive EVs attributes as interesting, pleasurable, positive and fun, which can lead to higher intention to purchase EVs.

### Theoretical contributions

Previous studies on EVs usually used self-interest orientation theories (e.g., theory of reasoned action, theory of planned behavior, value-attitude-behavior) to predict consumer purchase attitude and behavior (Lim et al., 2019; Wang et al., 2022b). Those studies are highly reliant on the expected costs and benefits of alternatives, such as time, money, peer influence and many others. Thus, they ignored the novel attributes of new products or services (e.g., EVs). This study was among the first that empirically validated and tested the significant relationships of CE, CK, PV, PU, PEU, attitude and intention toward the purchase of EVs based on the TAM model. Therefore, by investigating the influence of CE, CK and PV using the TAM model, this study will offer an alternative and a more comprehensive perspective on EVs purchasing behavior.

CE is still an underestimated predictor for explaining consumer purchase behavior in the marketing literature (Wang et al., 2021). Specifically, studies on the relationship between CE, PV and CK are scarce when compared to conventional studies, although a few empirical studies provided some explanation for establishing such relationships (Thomas et al., 2020). This study confirmed that CE positively and significantly influenced PV and CK. In other words, consumers who are of highly ethnocentric orientation are more willing to accept local products (i.e., EVs) even if those products may not be as good as imported products (e.g., quality, performance, attribute). They are more open to obtaining knowledge about local products and services compared to foreign ones. Thus, the influence of CE on consumer purchase attitude and behavior should be further investigated in future studies.

The preference of individuals for products or services is highly affected by their PV (Jan et al., 2019). There is a debate on the dimensions of PV to apply in marketing literature. For example, Sheth et al. (1991) mentioned that the social value, functional value, emotional value, epistemic value and conditional value should be used as the dimensions of PV. Other studies indicated that it is not necessary to include all of the five dimensions in marketing research due to certain drawbacks or weaknesses of conditional value and epistemic value (Caber et al., 2020; Rasoolimanesh et al., 2020). This study demonstrated that social value, functional value and emotional value can adequately represent PV which significantly influenced consumers’ PU and PEU toward purchasing EVs. This result provided a basis for future research in replicating the multidimensional concept of PV in determining consumers’ novelty products (e.g., EVs) purchasing behavior.

Researchers generally recognized that there is a positive relationship between CK and purchase attitude or intention (Wang et al., 2021). Nevertheless, the indirect effect of CK on attitude or intention has rarely been investigated in the literature. This study enriches the understanding of how CK influences PV, PU and PEU, which ultimately, influences the purchasing attitude and intention toward EVs. Consumers who are highly educated are more likely to recognize the advantages of new technology-related products or services and the results of this study also demonstrated that more knowledgeable consumers are more ready to adopt innovative green technology and pro-environmental products and services (i.e., EVs).

Lastly, many studies confirmed that PU and PEU have a separate influence on the attitude and intention under the TAM model (Wu et al., 2019; Jaiswal et al., 2021b). However, there is still room for researchers to further investigate the interrelationship between PU and PEU in EVs literature. Certain researchers suggested that PEU can influence consumer intentions to choose EVs technology directly or indirectly through PU on attitude and intention (Wang et al., 2017; Wang Y. et al., 2020). The following research showed that PEU has a positive influence on PU, which means that new technologies-related products or services should not only be considered for their ease of use but their products and services attribute of usefulness can also be easily assessed when they are easy to operate.

### Practical implications

There were several practical implications for EV operators based on the findings of this study. First, CE definitely plays a significant role in determining their PV and acceptance of knowledge about EVs. The EVs companies in China should highlight their local product attributes in their advertising messages, such as easy availability of locally made spare parts, little or no significant differences between local EVs performance (e.g., acceleration, battery efficiency) and foreign EVs, and of course, evoking their sense of patriotism and ethnocentrism when purchasing a Chinese-made product. Hence, Chinese people will perceive that purchasing local EVs will contribute to the creation of employment and the rebuilding of the Chinese manufacturing industry.

Our results showed that Chinese consumers are concerned about certain internal and external values about EVs. The feelings of using EVs are important for EVs users; they not only expect to have a good experience visiting EVs showrooms, but they also need to feel that driving EVs should be exciting and enjoyable. They need to be reassured that their decision-making process is a wise choice which can increase the protection of environment. Thus, the EV industries should emphasize the new technologies used in EVs and how the technology is constantly evolving. At the same time, they can highlight the emotional aspects of driving (acceleration, excitement) EVs which are the key determinants in selecting EVs. Finally, providing excellent pre-sale and after-sale service is another critical strategy to attract and maintain potential customers for EVs companies. Since the Chinese government is still providing incentives to EV buyers, including tax exemption, free license plates, etc., the EV companies should remind customers that they can access those benefits if they make their purchases early. They should also continue to emphasize the pro-environmental aspects of their purchasing behavior, such as helping to preserve natural resources and the reduction in greenhouse gas emissions through the purchase of EVs.

As PU and PEU were found to significantly influence attitude, and PEU was found to significantly influence PU, it is important to recognize that consumers’ perceived ease of driving EVs can significantly influence their perceptions about EVs’ usefulness (e.g., improve travel efficiency, improve living quality, reduce household expenditure, relieve physical and mental fatigue, protect the environment) which leads to a positive attitude toward a purchase decision. Thus, the EV operators should clearly explain the benefits of driving/using EVs (e.g., comfort, ease, etc.) when compared to traditional fuel vehicles. Finally, as specified in the past studies, a more positive attitude will impact consumers’ intentions and behaviors (Wang et al., 2022a,b). The results of this study demonstrated that the impact of attitude is noteworthy in affecting their purchase intention to purchase EVs. This implies that Chinese customers with more positive attitudes will more likely intend to buy EVs. Thus, EV companies are energized to spread EVs information and viewpoint *via* traditional and digital communication channels focusing on creating and maintaining a positive image of EVs for potential customers.

### Limitations

This study has certain limitations. First, the cross-sectional studies were inconclusive on the causality of relationships in this study. Second, this study was conducted inside the restricted scope of the Xuzhou city of Jiangsu territory, China, which cannot be generalizable to the entire EVs buyers’ population. Future studies should be conducted in other counties to see whether the findings can be replicated in other geographic regions. Furthermore, this study examined consumer purchase intention toward EVs based on the TAM model instead of being based on the actual purchase behavior itself. However, it should be noted that an individual’s actual purchase behavior is not equivalent to the estimated behavioral intention. For future research, it is necessary to undertake a comparative study on the influence of antecedent variables on consumers’ perceptions and behaviors toward domestic and imported EVs since this study only focuses on domestic EVs.

## Data availability statement

The raw data supporting the conclusions of this article will be made available by the authors, without undue reservation.

## Ethics statement

Ethical review and approval was not required for the study on human participants in accordance with the local legislation and institutional requirements. Written informed consent from the patients/participants OR patients/participants legal guardian/next of kin was not required to participate in this study in accordance with the national legislation and the institutional requirements.

## Author contributions

LW contributed to the design of the work, data analysis and interpretation, critical revision of the article, and final approval of the version to be published. Z-XW contributed to the design of the work, data interpretation, drafting the article, and final approval of the version to be published. AJ contributed to the design of the work. R-XW and M-RC contributed to the data analysis and interpretation. YG contributed to the drafting the article, data collection, and interpretation. PW contributed to the critical revision of the article, and final approval of the version to be published. All authors contributed to the article and approved the submitted version.
